# 具有不同酶活性并可抵抗特异shRNA降解的nm23-H1真核表达载体的构建和表达

**DOI:** 10.3779/j.issn.1009-3419.2014.03.01

**Published:** 2014-03-20

**Authors:** 战胜 鲁, 丽丽 郭, 林 李, 志浩 吴, 清华 周

**Affiliations:** 300052 天津，天津市肺癌转移与肿瘤微环境重点实验室，天津市肺癌研究所，天津医科大学总医院 Tianjin Key Laboratory of Lung Cancer Metastasis and Tumor Microenvironment, Tianjin Lung Cancer Institute, Tianjin Medical University General Hospital, Tianjin 300052, China

**Keywords:** Nm23-H1, 重叠延伸PCR, 定点突变, Western blot, Nm23-H1, Overlap extension PCR, Site-directed mutagenesis, Western blot

## Abstract

**背景与目的:**

已有的研究证明*nm23-H1*基因是一个重要的肿瘤转移抑制基因，但其抑制肿瘤转移的生化机理尚不完全清楚。*Nm23-H1*基因结构和功能异常与肿瘤的侵袭转移有密切关系。我们前期已构建了nm23-H1的短发夹RNA（short hairpin RNA, shRNA）载体以及可抵抗此shRNA降解的nm23-H1的cDNA的表达载体，在此基础上我们欲应用基因定点突变技术构建具有不同酶活性并能抵抗此shRNA降解的nm23-H1cDNA真核表达载体，并通过恢复实验验证其表达，为进一步研究肿瘤抑制基因*nm23-H1*的分子机制提供理论基础和实验依据。

**方法:**

以pcDNA3.1(+)-shRNA-resistant-nm23-H1质粒为突变模板，应用重叠延伸PCR方法引入*nm23-H1*基因四个单点突变和一个联合位点突变，并将突变基因片段克隆到真核表达载体pcDNA3.1Hygro(+)。将突变质粒转染人肺腺癌细胞株A549/nm23-H1-shRNA（稳定沉默*nm23-H1*基因），利用Western blot技术验证不同突变体nm23-H1蛋白的表达。

**结果:**

成功构建了shRNA抵抗的nm23-H1^S44A^、nm23-H1^P96S^、nm23-H1^H118F^、nm23-H1^S120G^、nm23-H1^P96S-S120G^五个突变型真核表达载体，经DNA序列分析突变的碱基序列与实验设计完全一致，经Western blot验证nm23-H1蛋白表达正常。

**结论:**

成功构建了五个具有不同突变位点的shRNA抵抗的*nm23-H1*基因真核表达载体，并且突变蛋白质nm23-H1表达正常，同时也表明重叠延伸PCR技术是一种高效、便捷、经济的DNA定点突变方法。

## Abstract (en)

**Background and objective:**

It has been proven that *nm23-H1* gene was a tumor metastatic suppressor gene. However, it's molecular mechanism of suppressing metastasis remains unexplored. There is a closely relationship between the abnormality of stucturs and functions of *nm23-H1* gene, and cancer invasion and metastasis. We have constructed the vector with nm23-H1-shRNA and the vector with nm23-H1cDNA resistant to the specific shRNA. So, we plan to construct shRNA-resistant eukaryotic expression vector of *nm23-H1* gene by site-directed mutagenesis, rescue experiment was performed to verify the *nm23-H1* gene expression, and to provide basement for studying the biochemical mechanisms of *nm23-H1* gene.

**Methods:**

Site-directed mutagenesis of *nm23-H1* gene was performed by overlap extension PCR method. Pure plasmid containing gene of *nm23-H1* (shRNA-resistant) was prepared. The desired five mutations were constructed and cloned into the eukaryotic vector pcDNA3.1Hygro(+). The human lung adenocarcinoma cell A549/nm23-H1-shRNA (stable *nm23-H1* gene silencing) was transfected with the five mutants, and the expression of the mutant proteins was determined by Western blot.

**Results:**

Five eukaryotic expression vectors (shRNA-resistant) of nm23-H1, nm23-H1^S44A^, nm23-H1^P96S^, nm23-H1^H118F^, nm23-H1^S120G^, nm23-H1^P96S-S120G^, were successfully constructed. The results of DNA sequencing confirmed that the base sequences of the genes were completely concordant with experiment design. The expression of nm23-H1 mutant proteins was verified by Western blot.

**Conclusion:**

Five eukaryotic expression vectors (shRNA-resistant) of *nm23-H1* gene were successfully constructed, and the mutant proteins were verified. The site-directed mutagenesis technical of overlap extension PCR is a efficient, simple and economical method.

*Nm23-H1*基因是第一个被发现的重要的肿瘤转移抑制基因，为*nm23*基因家族8个成员之一^[1, 2]^，该基因与人类多种肿瘤的侵袭转移密切相关。已有的研究表明：nm23-H1具有多种酶的活性^[3-6]^，其44位丝氨酸的磷酸化水平与其肿瘤转移抑制功能有关；118位的组氨酸突变为苯丙氨酸，丧失核苷二磷酸激酶活性，但不影响其肿瘤抑制功能；96位的脯氨酸突变为丝氨酸和120位的丝氨酸突变为甘氨酸则保留了核苷二磷酸激酶活性，丧失了组氨酸依赖的蛋白磷酸转移酶活性，其不同特定碱基的突变改变了其蛋白酶活性并影响到肿瘤的生物学行为。为此，本研究应用重叠延伸PCR方法构建shRNA（NM_000269.x-99s1c1:GCGTACCTTCATTGCGATCAA）抵抗的nm23-H1^S44A^、nm23-H1^P96S^、nm23-H1H118F、nm23-H1^S120G^、nm23-H1^P96S-S120G^五个突变型真核表达载体并验证其表达，从而为进一步揭示*nm23-H1*基因在肿瘤信号传导通路的分子机制奠定基础。

## 1 材料和方法

### 1.1 材料

pcDNA3.1Hygro(+)由本实验室保存；菌种大肠杆菌DH5α购自北京全式金生物技术有限公司；pcDNA3.1(+)-shRNA-resistant-nm23-H1由本实验室构建；PCR反应试剂盒购自Roche公司；Goldview购自北京赛百盛公司；质粒提取试剂盒、PCR产物纯化试剂盒、凝胶回收试剂盒、DNA连接试剂盒均购自QIAGEN公司；限制性内切酶*Bam*H1、*Xba*1购自NEB公司；核酸Marker购自美国Fermentas公司；nm23-H1鼠单抗、β-actin鼠单抗购自CST公司；其他试剂均为国产或进口分析纯；引物合成及DNA测序均由华大基因公司完成。

### 1.2 方法

#### 1.2.1 质粒提取

摇菌扩增质粒，HiSpeed Plasmid Midi Kit提取质粒，紫外分光光度仪测定质粒浓度及纯度。

#### 1.2.2 引物设计

包括侧翼引物和突变引物（表 1和表 2），根据GenBank报道的nm23-H1cDNA序列号（X17620）、重叠延伸PCR定点突变原理、*nm23-H1*基因shRNA抵抗突变碱基序列及载体pcDNA3.1Hygro(+)图谱，利用Oligo7软件共设计1对侧翼引物及4对突变引物。侧翼引物上游酶切位点*Bam*H1:GGATCC，下游酶切位点*Xba*1:TCTAGA；4个突变点分别为44位的丝氨酸（TCC）突变为丙氨酸（GCC）S44A，96位的脯氨酸（CCT）突变为丝氨酸（TCT）P96S，118位的组氨酸（CAT）突变为苯丙氨酸（TTT）H118F，120位的丝氨酸（AGT）突变为甘氨酸（GGT）S120G。

**1:** 侧翼引物 Outside primers

Primer name	Primer sequence(5’→3’)
F	(GC)GGATCCATGGCCAACTGTGAGCGAA
R	(CG)TCTAGATCATTCATAGATCCAGTTCTGA
Protective bases in brackets, the restriction sites were underlined.	

**2:** 突变引物 Mutation primers

Primer	name Primer sequence(5’→3’)
Fm44	GAAATTCATGCAAGCTG(T)CCGAAGATCTTCTCAAGG
Rm44	CCTTGAGAAGATCTTCGGC(A)AGCTTGCATGAATTTC
Fm96	CGGGGAGACCAACT(C)CTGCAGACTCCAAGC
Rm96	GCTTGGAGTCTGCAGA(G)GTTGGTCTCCCCG
Fm118	CAAGTTGGCAGGAACATTATATT(CA)TGGCAGTGATTCTGTGGAGA
Rm118	TCTCCACAGAATCACTGCCAAA(TG)TATAATGTTCCTGCCAACTTG
Fm120	GGAACATTATACATGGCG(A)GTGATTCTGTGGAGAGT
Rm120	ACTCTCCACAGAATCACC(T)GCCATGTATAATGTTCC
Normal bases in brackets, the bases for substitutions were underlined.	

#### 1.2.3 突变反应

每个待突变位点需要3个PCR反应来完成。首先以质粒pcDNA3.1(+)-shRNA-resistant-nm23-H1为模板，正向侧翼引物F和突变引物Rm为上下游引物进行PCR扩增（PCR1），扩增突变位点及其上游序列的DNA片段，产物命名P1，同时也以质粒pcDNA3.1(+)-shRNA-resistant-nm23-H1为模板，以突变引物Fm和反向侧翼引物R为上下游引物进行PCR扩增（PCR2），扩增含突变位点及其下游序列的DNA片段，产物命名为P2。最后取P1和P2各1 μL为模板，以正、反向侧翼引物F和R为上下游引物进行第3次PCR扩增（PCR3），用外侧引物将P1和P2拼接而成含突变位点的目的产物。PCR反应总体积均为25 μL：10×Buffer 2.5 μL，Taq DNA polymerase 0.5 μL，dNTPs（10 mM）1 μL，正、反向引物（20 μM）各0.5 μL，模板质粒pcDNA3.1(+)-shRNA-resistant-nm23-H1（230 ng/μL）0.5 μL，模板P1、P2各1 μL，余用双蒸水补足。PCR循环条件均为94 ℃预变性2 min；94 ℃变性30 s，60 ℃退火30 s，72 ℃延伸45 s，共30个循环；末次循环后，72 ℃再延伸8 min，4 ℃终止循环。用PCR产物纯化试剂盒将第3次PCR循环结束后产物纯化，经1%琼脂糖凝胶电泳鉴定（图 1），与目的片段大小（459 bp）相符，在nm23-H1^H118F^片段泳道可见一杂带，应用NDA凝胶回收试剂盒将杂带去除。

**1:**
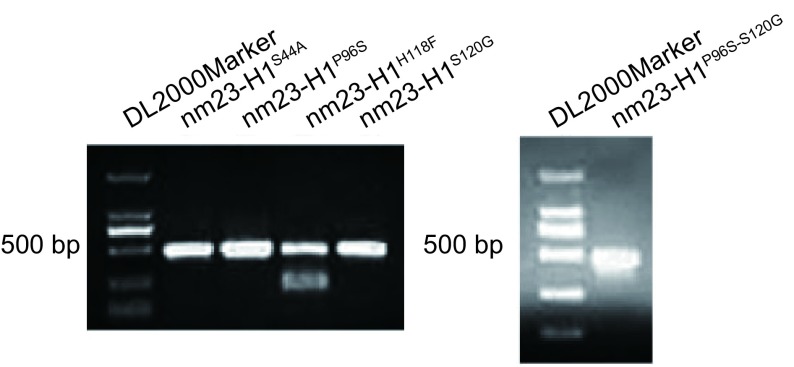
PCR结果 PCR amplification products were obtained by an overlap extension PCR

#### 1.2.4 联合位点（P96S-S120G）突变方法

以片段大小相符的nm23-H1^P96SPCR^纯化产物为模板进行重叠延伸PCR反应，操作步骤同前。

#### 1.2.5 目的基因片段与载体连接克隆

将载体pcDNA3.1Hygro(+)及PCR纯化产物进行*Bam*H1、*Xba*1双酶切反应，酶切产物再次纯化，以DNA连接试剂盒连接过夜。连接产物转化感受态DH5α大肠杆菌。取200 μL菌液涂布于含100μg/mL氨苄青霉素的LB平板中，37 ℃摇床过夜，挑取阳性菌落小摇。

#### 1.2.6 转染及Western blot验证nm23-H1蛋白表达

脂质体法将shRNA抵抗*nm23-H1*基因重组突变质粒转染稳定沉默*nm23-H1*基因表达的肺癌细胞株A549/nm23-H1-shRNA，48 h后细胞裂解后提取总蛋白，测定浓度后进行12%SDS-PAGE电泳。100 V转膜1 h，抗nm23-H1和β-actin一抗4 ℃孵育过夜，洗膜后室温二抗孵育1 h，ECL显影。

## 2 结果

### 2.1 突变载体菌液PCR鉴定

取菌液1 μL为模板，以侧翼正、反向引物为上下游引物进行PCR扩增反应，产物行1%琼脂糖凝胶电泳检测，所得片段与目的基因大小（459 bp）相符（图 2）。

**2:**
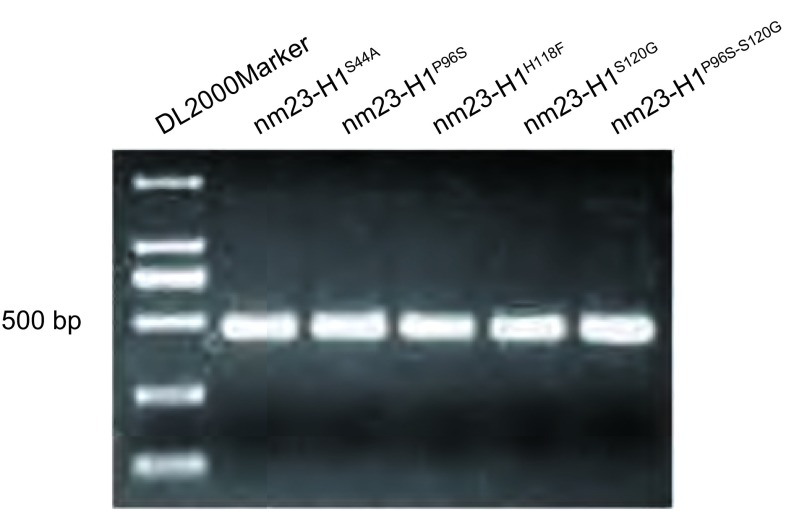
菌液PCR电泳结果 PCR amplification products from the bacteria

### 2.2 突变载体DNA测序结果

构建的4个单点突变型和1个联合位点突变型抗shRNA *nm23-H1*基因，经DNA测序验证，碱基突变结果与预期完全一致，抗shRNA碱基序列未发生改变（图 3）。

**3:**
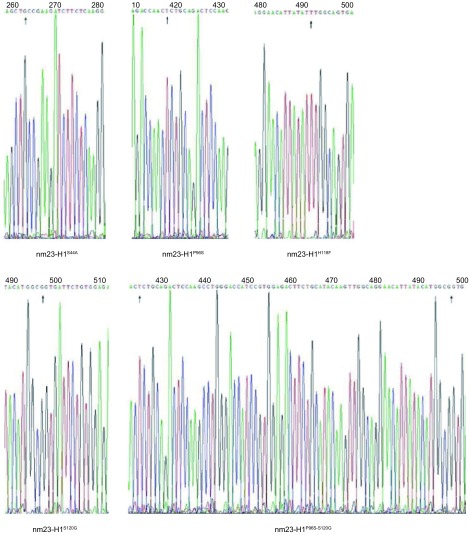
测序结果 Results of sequencing

### 2.3 shRNA抵抗*nm23-H1*基因重组突变质粒转染恢复实验

将shRNA抵抗*nm23-H1*基因重组突变质粒转染稳定沉默*nm23-H1*基因表达的肺癌细胞株A549/nm23-H1-shRNA，48 h后收获细胞蛋白作Western blot检测，显示恢复了nm23-H1蛋白的正常表达（图 4）。

**4:**
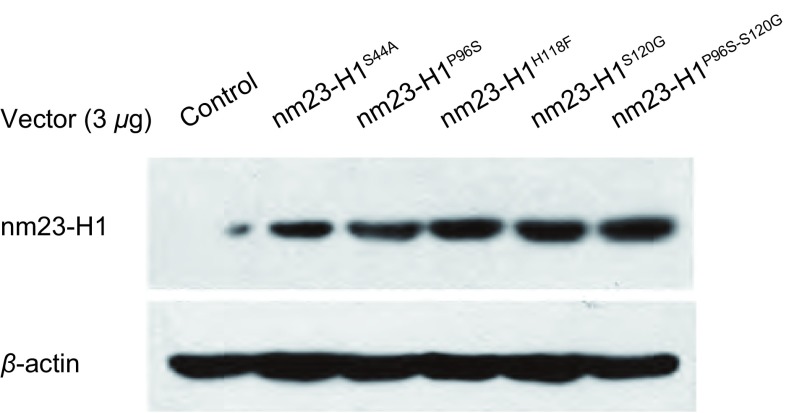
A549/nm23-H1-shRNA细胞基因重组质粒恢复实验，与对照组相比*nm23-H*基因重组质粒转染组重现nm23-H1蛋白的正常表达 shRNA rescue experiment in A549/nm23-H1-shRNA cells analyzed by Western blot

## 3 讨论

基于PCR方法的定点突变是指在基因的特定位点引入突变，有目的改变DNA序列中的碱基，是研究基因蛋白质结构与功能之间关系的有力工具^[7, 8]^。重叠延伸PCR技术采用互补引物，使PCR产物之间形成重叠链，从而在随后的扩增反应中通过重叠链的延伸将不同来源的扩增片段拼接起来，其成功的关键在于突变引物的设计，重叠部分应设计20个碱基左右，并且使引物的Tm值彼此接近。此技术利用PCR可以在体外进行有效的基因重组，并可在侧翼引物加入限制性酶切位点，可以将突变成功的基因片段克隆到目的载体上，比依靠试剂盒内切酶消化^[9]^的基因突变方法灵活、经济，后者需要事先克隆载体且后期筛选假阳性率高。重叠延伸PCR几乎没有任何特殊条件的限制，而且成功率很高，可利用这一技术很快获得其他依靠内切酶消化的方法难以得到的产物，因此运用非常广泛。本实验即是利用该技术通过设计突变引物和侧翼引物，并在侧翼引物加入酶切位点成功克隆到四个单点定点突变载体和一个联合位点突变载体，经验证与预期相符。

*Nm23*基因是美国国立癌症研究所的Steeg^[1]^等于1988年用消减杂交分离的方法筛选7个K-1735小鼠黑色素瘤细胞株时发现并分离出来的cDNA克隆，显示在2个低转移细胞株中其mRNA表达较另外5个高转移细胞株高10倍，随后在蛋白水平的检测中也有类似发现。*Nm23*基因是一个具有高度同源性的保守基因家族，广泛存在于细菌、酵母、植物、果蝇、鼠及人类在内的众多生物物种之中。迄今人类*nm23*基因家族已经发现8个成员，即nm23-H1至nm23-H8。其中nm23-H1亚型与人类肿瘤的发生、发展的关系最为密切，其mRNA由533个核苷酸组成，产生的蛋白质含有152个氨基酸，编码核苷二磷酸激酶-A（NDPK-A），分子量约为17 kDa，该基因具有抑制肿瘤转移潜能而不影响肿瘤大小的能力^[10]^。

侵袭转移是恶性肿瘤的重要生物学特征，也是导致肿瘤患者死亡的主要原因。*Nm23-H1*是第一个被发现的重要的肿瘤转移抑制基因，其抑制肿瘤侵袭转移的能力在多种肿瘤细胞实验中得到了证实，因此其抑制肿瘤侵袭转移作用的分子机理研究得到了持续的关注。同样在肺癌的基础研究中，*nm23-H1*基因被证明起着“转移抑制级联”^[11, 12]^的作用，作为靶基因通过级联效应调节下游基因的表达从而抑制肺癌细胞的侵袭转移表型，精细调控着细胞的多种生理功能，但其在抑制肺癌侵袭转移能力方面的分子机制尚未阐明。

本实验室研究工作已经证明肿瘤转移抑制基因*nm23-H*低表达、杂合性缺失和突变与肺癌的高转移性和预后不良密切相关，其编码蛋白核苷二磷酸激酶-A的酶活性可能在抑制肺癌侵袭转移方面起着重要作用。而已有的研究^[11-19]^表明*nm23-H1*基因编码蛋白具有3′→5′核酸外切酶、核苷二磷酸激酶和组氨酸蛋白激酶等多种酶的活性，并且相关研究显示，突变的*nm23-H1*基因蛋白在细胞的生长、分化及信号传导方面具有多种功能，其44位丝氨酸的磷酸化水平与其肿瘤转移抑制功能有关；118位的组氨酸突变为苯丙氨酸，丧失核苷二磷酸激酶活性，但不影响其肿瘤抑制功能；96位的脯氨酸突变为丝氨酸和120位的丝氨酸突变为甘氨酸则保留了核苷二磷酸激酶活性，丧失了组氨酸依赖的蛋白磷酸转移酶活性。本研究通过设计五个定点突变来改变其上述的相关酶活性，以shRNA抵抗的nm23-H1cDNA为模板利用重叠延伸PCR技术构建了shRNA抵抗的四个单点突变载体和一个联合位点突变载体，经鉴定符合预期要求，并验证了其蛋白表达，为下一步转染shRNA抵抗的A549肺癌细胞株^[17]^研究*nm23-H1*基因在肿瘤转移抑制及细胞信号传导中的生化机制奠定了基础。
